# Risk factors for ventilator-associated pneumonia in a surgical ICU

**DOI:** 10.1186/cc12077

**Published:** 2013-03-19

**Authors:** A Kundakci, O Ozkalaycı, P Zeyneloglu, H Arslan, A Pirat

**Affiliations:** 1 Baskent University Hospital, Ankara, Turkey

## Introduction

Ventilator-associated pneumonia (VAP) is the most common nosocomial infection in ICU patients who require mechanical ventilation support. The aim of this study was to determine predictors for the development of VAP in surgical ICU patients admitted to Baskent University Hospital.

## Methods

Following Institutional Review Board approval we performed this retrospective study, including 876 patients admitted to the surgical ICU between January 2009 and July 2012. After completing a review of patient data, 45 patients diagnosed with VAP were compared with 26 appropriate matches who were not. Patients' demographical features (age, sex, body weight), underlying diseases, etiology for ICU admission, APACHE II and Sequential Organ Failure Assessment scores, duration of hospitalization, organ dysfunctions, fluid balances, laboratory values, use of vasopressors, mechanical ventilation, nutrition, antibiotics, transfusions, features related to central venous catheterization, urinary catheterization, and intubation were the recorded parameters. Patients who were not intubated and were discharged or died within 2 days of ICU admission were excluded.

## Results

Out of 71 patients who were included in the final analysis, 45 patients (63%) had VAP. Comparing with the control group, patients who developed VAP were more likely to have diabetes mellitus and immunosuppression (*P *= 0.020 and *P *= 0.014, respectively). These patients also had higher APACHE II scores (*P *= 0.020) and a longer duration of mechanical ventilation (*P <*0.001), and more frequently had an open wound (*P *= 0.001). Following regression analysis, presence of diabetes mellitus (OR: 12.048; 95% CI: 1.157 to 125.000; *P *= 0.037), immunosuppression (OR: 16.949, 95% CI: 2.463 to 111.111; *P *= 0.004), and open wound (OR: 5.714; 95% CI: 1.017 to 37.258; *P *= 0.048), higher APACHE II scores (OR: 1.132; 95% CI: 1.022 to 1.254; *P *= 0.018), and prolonged duration of mechanical ventilation (OR: 1.084; 95% CI: 1.002 to 1.171; *P *= 0.043) were determined as risk factors for VAP. Fourteen-day and 28-day mortality rates for VAP were 19% and 29%, respectively (*P *= 0.760 and *P *= 1.000, respectively).

## Conclusion

The presence of diabetes mellitus and immunosuppression, higher APACHE II scores, longer mechanical ventilation duration, and presence of open wounds were predictors of VAP.
